# Developing clinical practice guidelines: types of evidence and outcomes; values and economics, synthesis, grading, and presentation and deriving recommendations

**DOI:** 10.1186/1748-5908-7-61

**Published:** 2012-07-04

**Authors:** Steven Woolf, Holger J Schünemann, Martin P Eccles, Jeremy M Grimshaw, Paul Shekelle

**Affiliations:** 1 Department of Family Medicine and Center on Human Needs, Virginia Commonwealth University, Richmond, VA, USA; 2 Departments of Clinical Epidemiology and Biostatistics and of Medicine, McMaster University, Hamilton, Canada; 3 Institute of Health and Society, Newcastle University, Baddiley-Clark Building, Richardson Road, Newcastle upon Tyne NE2 4AX, UK; 4 Clinical Epidemiology Program, Ottawa Hospital Research Institute, Ottawa, ON, Canada; 5 Department of Medicine, University of Ottawa, Ottawa, ON, Canada; 6 RAND Corporation, Santa Monica, CA 90407, USA; 7 Veterans Affairs Greater Los Angeles Healthcare System, Los Angeles, CA 90073, USA

## Abstract

Clinical practice guidelines are one of the foundations of efforts to improve healthcare. In 1999, we authored a paper about methods to develop guidelines. Since it was published, the methods of guideline development have progressed both in terms of methods and necessary procedures and the context for guideline development has changed with the emergence of guideline clearinghouses and large scale guideline production organisations (such as the UK National Institute for Health and Clinical Excellence). It therefore seems timely to, in a series of three articles, update and extend our earlier paper. In this second paper, we discuss issues of identifying and synthesizing evidence: deciding what type of evidence and outcomes to include in guidelines; integrating values into a guideline; incorporating economic considerations; synthesis, grading, and presentation of evidence; and moving from evidence to recommendations.

## Background

Clinical practice guidelines (hereafter referred to as guidelines) are one of the foundations of efforts to improve healthcare. The modern age of guidelines began with a 1992 Institute of Medicine (IOM) report, which defined guidelines as ‘systematically developed statements to assist practitioner and patient decisions about appropriate healthcare for specific clinical circumstances’ [1]. In 1999, we authored a paper about methods to develop guidelines [2]. It covered: identifying and refining the subject area of the guideline; running guideline development groups; identifying and assessing the evidence; translating evidence into a clinical practice guideline; and reviewing and updating guidelines. Since it was published, the methods of guideline development have progressed both in terms of methods and necessary procedures and the broad context for clinical practice guidelines has changed.

To help users identify and choose guidelines there has been the emergence of guideline clearing houses (such as the US Agency for Healthcare Research and Quality (AHRQ) Guideline Clearing House, http://www.guideline.gov) that identify and systematically characterize guidelines on a number of domains and the development of robust guideline appraisal instruments such as the AGREE tool [3,4]. There has been the appearance of large-scale guideline production organisations both at a national level (such as the UK National Institute for Health and Clinical Excellence or Scottish Intercollegiate Guidelines Network) and a condition level (such as the Ontario Cancer Guideline Program). There have also been relevant reports (that some of us have participated in) for the World Health Organisation [5] and professional societies (Schünemann HJ, Woodhead M, Anzueto A, Buist AS, MacNee W, Rabe KF, Heffner J. *A guide for guidelines for professional societies and other developers of recommendations: an official American Thoracic Society (ATS) / European Respiratory Society (ERS) Workshop Report*; in preparation). Such organizations and those interested in producing and using guidelines now have a high profile society in the Guidelines International Network (http://www.g-i-n.net/). Against this background it seems timely to, in a series of three articles, update and extend our earlier paper on the methods of developing clinical practice guidelines. This series is based on a background paper [6] we prepared for the IOM report ‘Clinical Practice Guidelines We Can Trust’ [7].

In the first paper, we discussed target audience(s) for guidelines, identifying topics for guidelines, guideline group composition, and the processes by which guideline groups function and the important procedural issue of conflicts of interest. In this second paper, we move on to discuss issues of identifying and synthesizing evidence: deciding what type of evidence and outcomes to include in guidelines; integrating values into a guideline; incorporating economic considerations; synthesis, grading, and presentation of evidence; and moving from evidence to recommendations. In the third paper, we will discuss the issues of: reviewing, reporting, and publishing guidelines; updating guidelines; and the two emerging issues of enhancing guideline implementability and how guidelines approach dealing with patients with co-morbid conditions.

## Deciding what type of evidence and outcomes to include in guidelines

Guidelines typically consider different clinical questions including: the identification of risk factors for conditions; diagnostic criteria for conditions; prognostic factors with and without treatment; the benefits and harms of different treatment options; the resources associated with different diagnostic or treatment options; and patients’ experiences of healthcare interventions. Different study designs provide the most reliable types of evidence for these different questions. Addressing this has implications for the conduct (searching, appraising, and synthesizing stages) of knowledge syntheses being undertaken to inform guideline recommendations. Important principles at this stage of guideline development include the need for guideline developers to make explicit decisions at the outset of the analytic process regarding the specific questions to be answered and the outcomes to be assessed, to have a clear understanding of the analytic logic of the recommendations, to use this model for keeping the analytic work of the group ‘on track,’ to be explicit about the types of evidence or opinion that support each component of the analytic logic, and to transmit this information clearly to the reader in the rationale statement of the guideline. Any model that achieves these organizational principles serves the purpose of an analytic framework [8-13].

## Developing an analytical framework

The analytic framework of a guideline is a key element in guideline development. It is in this critical stage that a group defines which questions must be answered to arrive at a recommendation, which types of evidence and information are relevant to the analysis, and by what criteria that evidence will be evaluated. The analytic work encompasses the examination of scientific evidence, expert opinion, clinical experience, and other relevant information and the use of decision rules to translate that information into recommendations. The end product of the process is captured in the analytic logic of the guideline, the rationale for the recommendations.

## Defining the analytic framework

The first step is to define the key questions. What information is required by the group to arrive at a recommendation? It begins with defining the criteria that must be met to convince the group that a clinical behavior should be advocated. The potential options depend on the viewpoint of the group and the nature of the topic. Some groups base the decision on current practice patterns or on opinions drawn from consensus or clinical experience. Many groups base the decision on scientific evidence, but they often differ in how they define effectiveness. Benefits can be defined by various measures of morbidity and mortality. Some groups consider benefits alone, and others consider adverse effects, costs, and other outcomes. It is therefore important for guideline developers to be as explicit as possible in defining outcomes of interest. It is not enough to state that the practice should be ‘clinically effective.’ What specific outcomes need to be affected to arrive at a recommendation? The group should decide which health, intermediate, and surrogate outcomes will be considered.

A health outcome refers to direct measures of health status, including measures of physical morbidity (*e.g.*, dyspnea, blindness, weakness), emotional well-being, and mortality (*e.g.*, survival, life expectancy). Eddy defines these as ‘outcomes that people can experience (feel physically or mentally) and care about’ [8]. An intermediate outcome is an effect that leads to a health outcome, and a surrogate outcome is an effect that is equivalent to a health outcome or can act as its proxy. Intermediate and surrogate outcomes are often physiologic variables, test results, or other measures that do not qualify as health outcomes by themselves but that have established pathophysiologic relationships with these outcomes. For coronary angioplasty, the establishment of arterial patency is an intermediate outcome leading to the desired health outcome of preventing subsequent ischemia. Surrogate outcomes could include electrocardiographic changes as a surrogate for cardiac ischemia, serum creatinine concentration for renal insufficiency, and pulmonary function tests for obstructive pulmonary disease. Although intermediate and surrogate outcomes are clearly less persuasive indices than actual health outcomes, they are often used in the analytic process because they are frequently the only validated outcome measures available in existing research. Guideline developers should determine which of these outcomes must be affected to convince the group that the maneuver should be recommended.

**Figure 1:**
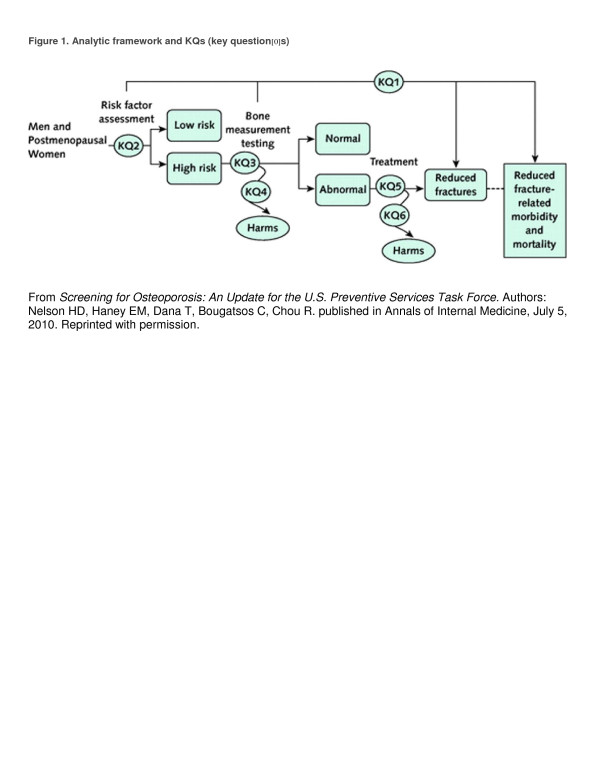
**Analytical framework and KQs (Keywords).** From *Screening for Osteoporosis: An Update for the U.S. Preventive Services Task Force.* Authors: Nelson HD, Haney EM, Dana T, Bougatsos C, Chou R. published in Annals of Internal Medicine, July 5, 2010. Reprinted with permission.

The potentially complex interrelationships between these outcomes are best visualized in a graphic or tabular format. A recent example of an analytic framework is shown in Figure 1, developed by the U.S. Preventive Services Task Force when considering a guideline about screening for osteoporosis [14]. This diagrammatic approach, first described in the late 1980s, emerged from earlier work on causal pathways [10], causal models [11], influence diagrams [12], and evidence models [13]. The construction of the diagram begins with listing the outcomes that the group has identified as important. This list of benefits, harms, and other outcomes reflects the key criteria that the group must address in its analytic process to assess appropriateness and arrive at a recommendation. Intermediate or surrogate outcomes that the group considers valid markers of effectiveness are next added to the diagram. The interconnecting lines, or linkages, that appear in Figure 1 represent the critical premises in the analytic logic that must be confirmed by the review process to support the recommendation. KQ1 is the overarching question—does risk factor assessment or bone measurement testing lead to reduced fracture-related morbidity and mortality? KQ2, KQ3, KQ4, KQ5, and KQ6 are questions about intermediate steps along the path, concerning the accuracy of risk factor assessment and bone measurement testing, potential harms of testing, and treatment of persons identified as abnormal.

The specification of the presumed relationship between intermediate, surrogate, and health outcomes in a visual analytic framework serves a number of useful purposes. It forces the analysts to make explicit, *a priori* decisions about the outcomes of interest to arrive at a recommendation. It allows others to judge whether important outcomes were overlooked. It makes explicit the group's opinions about the appropriateness of intermediate and surrogate outcomes as valid markers of health outcomes. The proposed interrelationships depicted in the diagram revealed the analysts’ assumptions about pathophysiologic relationships. They allow others to judge whether the correct questions were asked at the outset.

This type of analytic framework bears a visual resemblance to flowcharts, algorithms, and other graphics, but it differs importantly in content and purpose. The purpose of the visual analytic framework is prospective: the group defines at the outset the criteria it wishes to consider to arrive at a recommendation. The frameworks are conceptually different from algorithms. The linkages define the types of evidence to be reviewed and the outcomes represent measures of effectiveness, whereas the ‘arrows’ and outcomes in algorithms depict clinical choices, test results, or pathophysiologic events in the workup and treatment of patients [15,16].

## Filling in the evidence

The linkages in the visual analytic framework provide a ‘road map’ to guide the process of reviewing the evidence. They provide a specific list of questions that need to be answered by the group to arrive at a recommendation, though they do not define which types of evidence should be searched to provide the information. Once these questions have been answered, the literature review can proceed through an orderly process of searching admissible evidence to find support for the specific linkages in the analytic framework. The evidence supporting the linkages is often heterogeneous, with some linkages supported by randomized controlled trials and others supported by other classes of evidence.

Given the increasing availability of systematic reviews of different types of studies addressing different questions, guideline developers should initially search for relevant systematic reviews for each question as the availability of an up-to-date, high-quality, relevant systematic review could mitigate the need to undertake a systematic review *de novo*. Whitlock *et al.* provide guidance about the methodological and practical issues that developers need to consider when using existing systematic reviews in guideline development [17].

## Completing the analytic logic

Often, the information displayed in the analytic framework is only the starting point for more detailed analysis of the data. The completed diagram indicates only the class of evidence that supports a linkage and says little about the results of the studies, the consistency of the findings, or the quality of the data. Approaches for examining the evidence in more detail include the full range of analytic methods, such as simple narrative summaries, evidence tables, meta-analyses, and modeling. As a graphics device, the visual analytic framework is not meant to capture these details. Its role is to identify where the evidence sits in the analytic logic, not to describe what the evidence shows.

Writing a clear rationale statement is facilitated by the information in the analytic framework. The rationale statement can thereby summarize the benefits, harms, and other outcomes that were considered; why the outcomes were considered important (including consideration of patient preferences); the group's assumptions about the relationship between intermediate and surrogate outcomes and health outcomes; and the types of evidence that the group found in support of the linkages. If the review found linkages that lack supporting evidence, the rationale statement can speak honestly about the role that opinion, theory, or clinical experience played in arriving at a recommendation. This ‘truth in advertising’ helps ensure that the rationale statement provides clinicians, policymakers, and other guideline users with credible information about underlying assumptions. It also helps avoid misleading generalizations about the science, such as claiming that a maneuver is supported by ‘randomized controlled trials’ when such evidence supports only one linkage in the rationale. By sharing the blueprint for the recommendations, the linkages in the analytic logic allow groups to identify the pivotal assumptions about which they disagree.

Finally, by drawing attention to linkages that lack scientific support, the analytic framework highlights the most important outcomes to be examined by researchers to establish the effectiveness of a clinical practice. This information is essential, in an era of limited research funds, to set priorities and direct outcomes research toward the fundamental questions to be answered. The outcomes identified in the framework also provide a template for testing the efficacy of the guidelines themselves in research evaluating the effect of guidelines on quality of care.

## Integrating values in guideline development

Recommendations do not emerge directly from the empirical data reviewed by a guideline group. When the science clearly indicates substantial net benefit (benefit minus harms) or that an intervention is clearly ineffective or harmful, the need to consider values and preferences is less important. However, two major circumstances occur commonly in guideline development that require sensitivity to personal preferences and subjective judgments.

First, when the evidence is unclear, judgments about the occurrence and effect magnitude of an intervention often depend on subjective judgments about the quality of the studies. For example, a number of randomized controlled trials have evaluated the effectiveness of mammography screening for breast cancer, and a large body of empirical data about the effect size is available [18]. However, for two decades, experts with different opinions about the methods used in the trials have reached different conclusions about the quality of the evidence and the likely mortality reduction from mammography at different ages [19]. In the presence of scientific uncertainty, judgments based on other considerations often, and sometimes legitimately, take on greater importance. Guideline developers often consider clinical experience, expert opinion, the health condition in question and its severity, the potential harms of the intervention, and the potential harms of inaction. These judgments inevitably color how groups characterize the evidence and frame recommendations in the face of uncertainty [20]. In some instances, groups opt for neutrality, stating that there is insufficient evidence to make a recommendation [21]. In other circumstances, as when the condition poses great risk or there is little potential harm, the group may recommend the intervention despite inadequate evidence. In the opposite circumstance, when concerns about potential harms are heightened, a group may recommend against an intervention pending more convincing evidence [22]. Whatever choice is made, it is best for guideline developers to be transparent about value judgments [23]. The rationale for concluding that the evidence is strong, weak, or equivocal should be explained, preferably in detail. Concerns about the methods used for performing studies or evaluating outcomes should be outlined, both to explain the group’s rationale but also to guide future research to address limitations in the evidence. For example, knowing that guideline groups have recurrently cited contamination of the control group as a weakness in studies of an intervention will encourage future studies to devise innovative methods to address this concern.

Second, even when the occurrence or effect size is sufficiently clear from the data, the judgment of whether benefits outweigh harms can often be inherently subjective [24]. In such ‘close calls,’ people faced with the same data about the probabilities of benefits and harms can reach different conclusions about net benefit because of the different values, or utilities, they assign to the outcomes [25,26]. For example, the risk of developing urinary incontinence from radiation therapy for prostate cancer may be less disconcerting to an oncologist or patient who is focused on the hazard of the cancer than to a clinician or patient who is more concerned about quality of life than life expectancy. These subjective judgments are neither right nor wrong, but they do influence conclusions about net benefit and a group’s leanings on whether or not to recommend an intervention.

Groups have two options for dealing with close calls that involve difficult tradeoffs. First, they can make the decision themselves and conclude whether benefits outweigh harms. The group, with its in-depth knowledge of the clinical topic and the underlying science, can infer how most patients would react if faced with the same information. Such groups act as a proxy for patients, and the advantage of this approach is that the group has mastery of details that are often beyond the ability of most patients to digest or most busy clinicians to explain. The disadvantage of this approach is its inherent paternalism and the risk of misjudgments by the group [27]. The second option for dealing with close calls is for the group to eschew a blanket recommendation but to instead encourage shared or informed decision-making, in which the patient is encouraged to review the tradeoffs with their clinician and make an individual decision based on personal preferences [28,29]. When this is done, the group expressly avoids taking a policy stance. Its policy is to advise clinicians to engage the patient in the decision; such groups recognize that the determination of whether benefits outweigh harms is sensitive to utilities, a determination that can only be made individually by the patient and clinician, not by a guideline group [30]. The advantage of this approach is its respect for autonomy and individual choice, in which guidelines become a tool for patient empowerment, engagement, and activation [31,32]. If the group eschews a recommendation and instead advocates shared decision-making, it is helpful if the guideline includes details about the content areas the patient-clinician conversation should cover. The guideline group is likely to have a clear sense of the preference-sensitive issues that influence the benefit-harm tradeoff and can therefore outline the items the patient and clinician should review, the relevant evidence, the role of decision aids, and other suggestions for incorporating personal preferences into the decision-making process.

## Incorporating economic considerations in guideline development

There has been no widely accepted successful way of incorporating economic considerations into guidelines. However, the reasons for considering costs are clearly stated: ‘Health interventions are not free, people are not infinitely rich, and the budgets of [healthcare] programs are limited. For every dollar’s worth of healthcare that is consumed, a dollar will be paid. While these payments can be laundered, disguised or hidden, they will not go away’ [8]. Such opportunity costs are a universal phenomenon. It is also the case that while considerations of effectiveness may be applicable across different healthcare systems, considerations of cost and values are more likely to be healthcare system-specific. Therefore, a cost-effectiveness guideline may be less transferable than one based solely on clinical effectiveness.

In the USA, the 1992 IOM report [33] offered the aspirational recommendation that every set of clinical guidelines should include information on the cost implications of the alternative preventive, diagnostic, and management strategies for each clinical situation. The stated rationale was that this information would help potential users to better evaluate the potential consequences of different practices. However, they then acknowledged that ‘the reality is that this recommendation poses major methodological and practical challenges.’ Although there is emerging practical experience, this position has not really changed. In addition, it has also become recognized that issues of cost are much more likely to be health system-specific (as compared to the clinical evidence areas of guideline development) and so, unless explicitly mandated—like the UK National Institute for Health and Clinical Excellence (NICE)—many guideline developers do not do this.

Some guideline development organizations (*e.g.*, NICE) advocate the review of appropriate cost-effectiveness studies alongside the review of the clinical evidence, though, in their guideline development manual, they note that ‘only rarely will the health economic literature be comprehensive enough and conclusive enough that no further analysis is required. Additional economic analyses will usually be needed.’ The available ‘economic evidence’ may be limited in terms of general applicability to the specific context of the clinical guideline, but can be useful in framing the general bounds of cost-effectiveness of management options for a clinical condition and providing an explicit source for some of the assumptions that may have to be made.

The methods of incorporating economic considerations are shaped by the methods of guideline development [34]. Early on in the development of each of the guidelines, there is a fundamental decision to be made about how to summarize the data and whether or not there are common outcomes across studies. If common outcomes are available, then it may be possible to use quantitative techniques (meta-analysis or meta-regression) leading to summary relative and absolute estimates of benefit, and it may then be possible to formally combine the elements of effectiveness and cost into a summary cost-effectiveness statistic. With relatively broad clinical areas (*e.g.*, the management of type 2 diabetes), it is more difficult to do this, whereas for narrower areas (*e.g.*, choosing a drug to treat depression) it is may be more feasible.

If the evidence summary is to be qualitative (a narrative review of studies) the data can be set out in ways that facilitate easy comparison between studies by using common descriptors (*e.g.*, study design, study population, intervention, duration of intervention) using evidence tables. However, under these circumstances it may not be possible to make estimates of cost-effectiveness unless the evidence summary is dominated by one study with appropriate outcomes. For guidelines that use qualitative evidence summary methods (not amenable to meta-analysis), it is usually only possible to present cost data alongside the evidence of clinical effectiveness allowing a reader to make their own judgments about the relative weight to be ascribed to these two dimensions of evidence. It is possible to make cost minimization statements such as: ‘as the treatments appear equivalent clinicians should offer the cheapest preparation that patients can tolerate and comply with.’

For guidelines focused on a single decision, it may be possible to incorporate economic data into a formal decision analysis framework. Traditionally, it is the province of health economics to model (combine, adjust, extrapolate, represent) intermediate clinical outcome data and data from other sources to explore the overall costs and consequences of treatment alternatives. In principle, it is possible to map clinical data onto generic quality of life scores, model the advancement of disease and produce cost per quality-adjusted life year (QALY) estimates for each treatment decision. However, such a process contrasts with the above methods in a number of ways. First, although they may have a role in informing the questions, values, and assumptions that go into a model, there is no clear role for a multi-disciplinary guideline development group in deriving recommendations around the clinical decision—the ‘right decision’ is produced by the model. Second, the data are aggregated into a single metric, the constituent elements of which (and their associated uncertainty) are not transparent. Third, the complexity of modeling a single decision is often such that the viability of the method to deal with more complex clinical decisions, which have multiple interdependencies, has to be questioned. Therefore, the appropriate application of a decision analysis-driven guideline is currently unclear and a question for further research.

## Guideline recommendations

### Wording recommendations

An important aspect of developing recommendations that will favorably influence care is the wording used for the recommendations. McDonald [35] and others have lamented the existence of recommendations that are vague or nonspecific, and that use what they call ‘weasel words,’ as in ‘patients with < condition name > should be offered the intervention if clinically appropriate’ or ‘clinicians should follow-up patients given the intervention every four weeks, or sooner if necessary’ because clinicians trying to use the guideline may have difficulty, or themselves be uncertain about, what constitutes ‘clinically appropriate’ or ‘if necessary.’ Grol *et al.* found that Dutch general practitioners followed guideline recommendations that were vague or nonspecific 35% of the time, while ‘clear’ recommendations were followed 67% of the time [36]. An experimental study using vignettes of patients with back pain found that specific guidelines produced more appropriate and less inappropriate orders for electro-diagnostic tests than did vague guidelines [37]. Michie and Johnston, using evidence from psychological research, went so far as to conclude that the ‘most cost effective intervention to increase the implementation of guidelines is rewriting guidelines in behaviorally specific terms’ [38].

However, a standard for wording of recommendation does not exist [39]. The lack of a standard is reflected in the results of a comprehensive evaluation of over 1,275 randomly selected recommendations (out of over 7527) from the National Guideline Clearinghouse by Hussain *et al. *[40]. Recommendations were presented with great inconsistency within and across guidelines, and 31.6% did not present executable actions. Over one-half (52.6%) did not indicate the strength of the recommendation.

The Editorial Board of the National Guideline Clearinghouse ‘encourages [guideline] developers to formulate recommendation statements that are ‘actionable’ and that employ active voice, rather than passive voice’ [41]. In the UK, NICE describes that recommendations should be clear and concise, but include sufficient information that they can be understood without reference to other supporting material (National Institutes for Health and Clinical Excellence Handbook) [42].

Clarity and precision in guidelines are desirable not only to facilitate implementation by clinicians and patients but also to be incorporated into decision support tools (*e.g.*, prompts used by electronic medical records, standing orders, checklists) to facilitate guideline implementation. However, guideline developers who closely follow evidence-based methods in formulating guidelines may find the science inadequate to justify such precision. Under such circumstances, ambiguity may more faithfully reflect adherence to the data than would spurious precision. For example, the evidence indicates that Papanicolaou smears are effective every one to three years, and that mammographic screening can reduce mortality whether it is performed annually or every other year [43]. For some screening tests, there is inadequate evidence to specify any interval or to define the risk groups for which screening is appropriate. When research has not determined that one interval is effective and another is not, arbitrarily fabricating a precise answer may satisfy demands for ‘clear’ guidelines but it departs from the evidence. It also exposes clinicians and patients to potential harm by proscribing care practices that may be entirely reasonable. Evidence-based guideline developers therefore always struggle with the tension between providing guidance that is as clear and precise as possible and the need to not reach beyond the supporting science.

The little evidence that does exist suggests that consumers of healthcare recommendations prefer knowing about the underlying quality of evidence, and that symbols to indicate the strength of recommendations are more informative than numbers [44,45]. Based on their review of the NGC database, Hussain *et al.* suggest six criteria to be followed in the presentation and formulation of recommendations (Table 1).

**Table 1:** Criteria to be followed in the presentation and formulation of recommendations

1. Identify the critical recommendations in guideline text using semantic indicators (such as ‘The Committee recommends. . .’ or ‘Whenever X, Y, and Z occur clinicians should . . .’) and formatting (*e.g.*, bullets, enumeration, and bold face text).	
2. Use consistent semantic and formatting indicators throughout the publication.	
3. Group recommendations together in a summary section to facilitate their identification.	
4. Do not use assertions of fact as recommendations. Recommendations must be decidable and executable.	
5. Avoid embedding recommendation text deep within long paragraphs. Ideally, recommendations should be stated in the first (topic) sentence of the paragraph and the remainder of the paragraph can be used to amplify the suggested guidance.	
6. Clearly and consistently assign evidence quality and recommendation strength in proximity to each recommendation and distinguish between the distinct concepts of quality of evidence and strength of recommendation.	

### What approaches to grading the quality of evidence and strength of recommendations exist?

Grading of healthcare recommendations began with the Canadian Task Force on the Periodic Health Examination over three decades ago [46]. In 2002, AHRQ published a systematic review of existing systems to grade the quality of evidence and strength of recommendations [47]. The AHRQ review considered 40 systems until the year 2000 that addressed grading the strength of a body of evidence. The important domains and elements for the systems to grade the strength of evidence that the authors agreed on were quality (the aggregate of quality ratings for individual studies, predicated on the extent to which bias was minimized), quantity (magnitude of effect, numbers of studies, and sample size or power), and consistency (for any given topic, the extent to which similar findings are reported using similar and different study designs).

In 2005, the Canadian Optimal Medication Prescribing and Utilization Service (COMPUS), a department within the Canadian Agency for Drugs and Technology in Health (CADTH), used a detailed process to evaluate and select an evidence grading system and expanded the work by AHRQ (while accepting it) until the year 2005 [48]. Nearly 50 evidence grading systems were identified from 11 review articles. Experts in evidence evaluation methodology helped identify an additional 10 instruments or systems not included in the list of identified grading systems. The identified instruments and systems were evaluated using the AHRQ evaluation grids. The highest scoring instruments were the Grading of Recommendations, Assessment, Development and Evaluation (GRADE) working group and the SIGN approaches [48]. A second round of expert consultation and stakeholder input from all interested parties confirmed the selection of these instruments. However, SIGN—while providing a detailed system for assessing the quality of individual studies—provided no clear guidance for summarizing the quality of evidence across studies and for moving from the research evidence to recommendations. SIGN therefore recently adopted GRADE that laid out these steps more explicitly.

### GRADE

A number of publications describe the GRADE approach and its development [44,49-57]. The GRADE working group (http://www.gradeworkinggroup.org) [49] emphasizes the link between the quality of a body of evidence and the recommendation, but recognizes that other factors beyond the quality of evidence contribute to the strength of a recommendation, such as patient values and preferences [58,59].

GRADE considers eight factors in the assessments of the quality of evidence for each important outcome (Table 2). Concerns about any of five factors can lower the confidence in an estimate of effect and study quality: study design and execution (risk of bias); consistency of the evidence across studies; directness of the evidence (including concepts of generalizability, transferability and external validity); the precision of the estimate of the effect; and publication bias. The presence of any of the following three factors can increase the quality of evidence: a strong or very strong association; a dose-effect relationship; and all plausible residual confounding may be working to reduce the demonstrated effect or increase the effect if no effect was observed. The overall quality of evidence is determined by the lowest quality of evidence for each of the critical outcomes. However, when outcomes point in the same direction (all critical outcomes suggesting benefit), then the overall quality of evidence reflects the quality of the better evidence (*e.g.*, two critical outcomes showing convincing benefit are of low quality and a third of very low quality, the overall quality is not reduced from low to very low).

**Table 2:** A summary of the GRADE approach to grading the quality of evidence for each outcome

**Source of body of evidence**	**Initial rating of quality**	**Factors that may decrease the quality**	**Factors that may increase the quality**	Final quality of a body of evidence *
				
Randomised trials	High	1. Risk of bias	1. Large effect	High
		2. Inconsistency	2. Dose–response	(⊕⊕⊕⊕ or A)
		3. Indirectness	3. All plausible residual confounding would reduce the demonstrated effect or would suggest a spurious effect if no effect was observed	Moderate
		4. Imprecision		(⊕⊕⊕◯ or B)
Observational studies	Low	5. Publication bias		Low
				(⊕⊕◯◯ or C)
				Very low
					(⊕◯◯◯ or D)

*Quality of evidence definitions.

High: Further research is very unlikely to change confidence in the estimate of effect.

Moderate: Further research is likely to have an important impact on confidence in the estimate of effect and may change the estimate.

Low: Further research is very likely to have an important impact on confidence in the estimate of effect and is likely to change the estimate.

Very low: Any estimate of effect is very uncertain.

A substantial conceptual difference between GRADE and other approaches is the handling of expert opinion. GRADE specifically acknowledges that expertise is required for interpretation of any form of evidence (‘judgments’) but considers that opinion is an interpretation of—sometimes unsystematic—evidence, but not a form of evidence.

### Factors that influence recommendations

Four factors influence whether a panel makes a recommendation for or against a management strategy. These four factors include: the quality of the available supporting body of evidence; the magnitude of the difference between the benefits and undesirable downsides or harms; the certainty about or variability in values and preferences of patients; and the resource expenditure associated with the management options.

### Quality of evidence

The quality of evidence reflects the confidence or certainty in the estimates of effects related to an outcome. If guideline panels are uncertain of the magnitude of the benefits and harms of an intervention, it is unlikely they can make a strong recommendation for that intervention (see section on quality of evidence). Thus, even when there is an apparent large gradient in the balance of advantages and disadvantages, guideline developers will be appropriately reluctant to offer a strong recommendation for an intervention if the quality of the evidence is low.

### The balance between benefits and undesirable downsides

When the benefits of following the recommendation clearly outweigh the downsides, it is more likely that the recommendation will be strong. When the desirable and undesirable consequences are closely balanced, a weaker recommendation is warranted. While most original studies and systematic reviews present the magnitudes of effect of outcomes in relative terms (*e.g.*, relative risk, hazard ratio, odds ratio), weighing the magnitude of the difference between the benefits and downsides to develop a recommendation also requires the knowledge of the likely absolute effects for a specific population or situation. If the guideline panel judges that the balance between desirable and undesirable effects varies by baseline risk, it can issue separate recommendations for groups with different baselines risks when tools for risk stratification are available for the guideline users [60,61]. Often, when values and preferences or attitude towards the resource use may differ from those assumed by guideline developers, patients, clinicians, and policy makers may choose to examine the magnitude of effects of management options on the outcomes of interest themselves, rather than relying on judgments of those making the recommendation.

### Uncertainty or variability of patient values and preferences

Different patients can take different views about what outcome constitutes benefit or harm and clinicians’ understanding of importance of particular outcomes for patients can differ from that of the patients. Explicit consideration of patients’ values and preferences in making recommendations stems from acknowledgement of patients’ liberty (autonomy). Alternative management strategies always have associated advantages and disadvantages and thus a trade-off is always necessary. How patients and guideline panel members value particular benefits, risks, and inconvenience is critical to any recommendation and its strength. However, data about patients’ preferences and values are often limited. GRADE urges guideline panels to state explicitly what values and preferences they considered and what weight they placed on each outcome. This transparent explanation facilitates the interpretation of recommendations, especially weak ones for which the best course of action is less certain.

### Costs or resource utilization

One could consider resource utilization as one of the outcomes when balancing positive and negative consequences of competing management strategies. However, as was mentioned above, costs are much more variable over time and geographic areas than are other outcomes. In addition, the implications of the utilized resource vary widely. For example, a year’s prescription of a drug may pay for a single nurse’s salary in the United States, ten nurses’ salaries in Romania, and thirty nurses’ salaries in India. Therefore, while higher costs will reduce the likelihood of a strong recommendation in favor of a particular intervention, the context of the recommendation will be critical. In considering resource allocation, those making recommendations must be very specific about the setting to which a recommendation applies and the perspective they took, *i.e.*, that of a patient, a third party payer or society as a whole.

### Making recommendations

Those making recommendations may have higher or lower confidence that following their recommendation will do more good than harm across the range of patients for whom the recommendation is intended [62]. They inform users of guidelines (*e.g.*, clinicians, patients and their family members, policy makers) about the degree of their confidence by specifying the strength of recommendations. While in reality the balance between desirable and undesirable consequences is a continuum, the GRADE approach uses two grades of the strength of recommendations—strong or weak (also known as conditional)—reflecting the confidence in the clarity of that balance or lack thereof (Table 3). This dichotomy serves to simplify the message, and improve understanding and communication. In various guidelines following the GRADE approach words other than ‘weak’ have been used to express the lower confidence in the balance of benefits and downsides, *e.g.*, ‘conditional,’ ‘qualified,’ or ‘discretionary.’

**Table 3:** Implications of the two grades of strength of recommendations in the GRADE approach

**Target group**	Strong recommendations*	**Conditional (weak) recommendations**
Patients	Most people in your situation would want the recommended course of action and only a small proportion would not	The majority of people in your situation would want the recommended course of action, but many would not
Clinicians	Most patients should receive the recommended course of action	Recognise that different choices will be appropriate for different patients and that you must make greater effort with helping each patient to arrive at a management decision consistent with his or her values and preferences
		Decision aids and shared decision making are particularly useful
Policy makers and developers of quality indicators	The recommendation can be adopted as a policy in most situations	Policy making will require substantial debate and involvement of many stakeholders

* Strong recommendations based on high quality evidence will apply to most patients for whom these recommendations are made, but they may not apply to all patients in all conditions; no recommendation can take into account all of the often-compelling unique features of individual patients and clinical circumstances.

Sometimes, authors of guidelines formulate their recommendations only as statements about the available evidence (*e.g.*, chromones are effective in the treatment of allergic rhinitis), but do not explicitly specify what action should follow (*e.g.*, should chromones be used in treatment of allergic rhinitis, given all other available treatment options?) [40]. GRADE suggests phrasing recommendations in an active voice as clear indications what specific action should follow. For example, many guidelines developed following the GRADE approach worded their recommendations as ‘we recommend …’ and ‘we suggest …’ to distinguish strong from weak recommendations. Alternatives for strong recommendations include ‘clinicians should …’ while weak recommendations can be phrased as ‘clinicians might …’ or ‘we conditionally recommend ….’ Expressing the strength of recommendations may become even more challenging when they are formulated in languages other than English.

### Should guideline panels make recommendations in the face of very low-quality evidence?

In the face of very low-quality evidence, there is broad agreement that the option of not making a recommendation should be included for all guideline panels. However, higher-quality evidence may never be obtained, and physicians need guidance regardless of the quality of the underlying evidence. Ideally, guideline panels should use their best judgments to make specific and unambiguous recommendations (albeit conditional ones in the face of very low quality evidence) and transparently lay out the judgments they make. Some groups maintain that no recommendations should be made when the evidence is considered ‘insufficient.’ The USPSTF uses an ‘insufficient evidence to make a recommendation’ category. It is argued that it is too risky for a guideline panel to make a recommendation on low- or very low-quality when there is a substantial risk the panel may be wrong.

### Research recommendations

There are not well-established criteria for guiding panels to make the determination of whether research should be done. Nonetheless, the criteria in Table 4 must be met for a recommendation for use of interventions in the context of research to be sensible [63,64]. The research recommendations should be detailed regarding the specific research questions that should be addressed, particularly which patient-important outcomes should be measured, and other relevant aspects of what research is needed [65]. Because the target audience for most guidelines is clinicians, the recommendations for research may seem misplaced and distracting among the recommendations related to practice. If this is the case, research recommendations could be placed in an appendix or special sections in the guideline directed at researchers and research funding agencies. A similar format decision should affect the design of executive summaries.

**Table 4:** Criteria to be met for a recommendation for use of interventions in the context of research to be sensible

1. There must be important uncertainty about the effects of the intervention (*e.g.*, low or very low quality evidence for either or both the desirable and undesirable consequences.	
2. Further research must have the potential to reduce that uncertainty at a reasonable cost.	
3. The potential benefits and savings of reducing the uncertainty outweigh the potential harms and costs of either using or not using the intervention based on currently available evidence	

## Summary

In this paper, we have discussed the issues of identifying and synthesizing evidence: deciding what type of evidence and outcomes to include in guidelines; integrating values into a guideline; incorporating economic considerations; synthesis, grading, and presentation of evidence; and moving from evidence to recommendations. In the third and final paper in the series, we will discuss the issues of: reviewing, reporting, and publishing guidelines; updating guidelines; and the two emerging issues of enhancing guideline implementability and how guidelines approach dealing with patients with co-morbid conditions.

## Competing interests

MPE is Editor in Chief of Implementation Science; Jeremy Grimshaw is an Editorial Board member. All decisions on this paper were made by another editor. The authors have all been involved in guideline development for a range of different organizations. Holger Schunemann is, and Martin Eccles has been, a member of the GRADE Group.

## Authors’ contributions

All authors contributed to the writing of this article and approved the final draft.
